# What Medical Oncologist Residents Think about the Italian Speciality Schools: A Survey of the Italian Association of Medical Oncology (AIOM) on Educational, Clinical and Research Activities

**DOI:** 10.1371/journal.pone.0159146

**Published:** 2016-07-12

**Authors:** Anna Moretti, Michele Ghidini, Carmine De Angelis, Matteo Lambertini, Chiara Cremolini, Martina Imbimbo, Rossana Berardi, Massimo Di Maio, Stefano Cascinu, Nicla La Verde

**Affiliations:** 1 Department of Medical Oncology, Azienda Socio Sanitaria Territoriale Fatebenefratelli Sacco, Presidio Ospedaliero Fatebenefratelli, Milan, Italy; 2 Department of Medicine, Division of Oncology, Azienda Socio Sanitaria Territoriale Di Cremona, Presidio Ospedaliero di Cremona, Cremona, Italy; 3 Department of Clinical Medicine and Surgery, Oncology Division, University of Naples "Federico II", Naples, Italy; 4 Department of Medical Oncology, U.O. Oncologia Medica 2, IRCCS AOU San Martino-IST, Genova, Italy; 5 Polo Oncologico, Azienda Ospedaliero-Universitaria Pisana University of Pisa, Pisa, Italy; 6 Department of Medical Oncology, Fondazione IRCCS Istituto Nazionale Tumori, Milan, Italy; 7 Medical Oncology, Università Politecnica delle Marche Ospedali Riuniti di Ancona, Ancona, Italy; 8 Department of Medical Oncology, Università di Torino, SCDU Oncologia Medica, AOU San Luigi Gonzaga, Orbassano, Turin, Italy; 9 Department of Medical Oncology, Università degli Studi di Modena, Modena, Italy; National Institute of Health, ITALY

## Abstract

**Background and objectives:**

Relevant heterogeneity exists among Postgraduate Schools in Medical Oncology, also within the same country. In order to provide a comprehensive overview of the landscape of Italian Postgraduate Schools in Medical Oncology, the Italian Association of Medical Oncology (AIOM) undertook an online survey, inviting all the residents to describe their daily activities and to express their overall satisfaction about their programs.

**Methods:**

A team composed of five residents and three consultants in medical oncology prepared a 38 items questionnaire that was published online in a reserved section, accessible through a link sent by e-mail. Residents were invited to anonymously fill in the questionnaire that included the following sub-sections: quality of teaching, clinical and research activity, overall satisfaction.

**Results:**

Three-hundred and eleven (57%) out of 547 invited residents filled in the questionnaire. Two-hundred and twenty-three (72%) participants declared that attending lessons was frequently difficult and 153 (49%) declared they did not gain substantial improvement in their knowledge from them. Fifty-five percent stated that they did not receive lessons on palliative care. Their overall judgment about didactic activity was low in 63% of the interviewed. The satisfaction for clinical activity was in 86% of cases good: 84% recognized that, during the training period, they acquired a progressive independence on patients' management. About research activity, the majority (79%) of participants in the survey was actively engaged in managing patients included in clinical trials but the satisfaction level for the involvement in research activities was quite low (54%). Overall, 246 residents (79%) gave a positive global judgment of their Medical Oncology Schools.

**Conclusions:**

The landscape of Italian Postgraduate Schools in Medical Oncology is quite heterogeneous across the country. Some improvements in the organization of teaching and in the access to research opportunity are needed; the perception about clinical activity and the overall judgment of the programs are quite satisfactory.

## Introduction

Medical oncology is a relatively “*young specialty”*, born in the Sixties and having a quick development in the following 10–15 years. At the same time, the post-graduate school in Medical Oncology presents a great variability at the European level and also within each country [1].

The main scientific oncologic societies (American Society of Clinical Oncology and European Society of Medical Oncology [ESMO]) have recently demonstrated an interest in the quality of training of young oncologists. In 2014, ESMO published a position paper about the relevance of Medical Oncology and, consequently, the need for a high training profile [2]; moreover, the two international societies together have formulated a set of guidelines with a global perspective for the clinical training required for physicians to qualify as medical oncologists [3].

In Italy, the modality of training, as well as the didactic work and the research opportunities, differ across the country. This heterogeneity has become even more relevant in the era of multimodal approach for the management of patients and of fast expanding knowledge. In fact, in the multidisciplinary teams, medical oncologists play a key role both in choosing the most appropriate treatment options and in promoting therapeutic innovation through clinical and translational research [2]. Hence, this requires a dedicated attention to training and continuing education [4,5]. Two different surveys have recently explored the young oncologists background, focusing on their professional expectations and burnout level, both in Europe and in USA [6,7]. Interestingly, both these studies have demonstrated the need for attention to the training of young oncologists, not only regarding how they experience their formation period, but also how they evaluate the organization of their training school.

In this context, in order to obtain more comprehensive information, the Italian Association of Medical Oncology (AIOM) undertook an online survey on the landscape of medical oncology training within the country, which was anonymously sent to all the residents of the Italian Postgraduate Schools of Oncology.

The aim of this survey was to understand the residents’ point of view about several aspects of the School of Medical Oncology: educational activities, clinical activities (particularly about daily activities and work periods abroad), research activities and modality of final examination.

## Materials and Methods

A team composed of five residents and two consultants in medical oncology prepared a 38-item questionnaire to be submitted to all Italian Medical Oncology trainees. The questionnaire was anonymous, the participants were asked to fill in the gaps with data about sex, age and year of training. The first part of the questionnaire (22 questions) regarded common activities for all students. Questions evaluated learning activities such as lectures, examinations and complementary clinical experiences (e.g. training abroad). The second part of the questionnaire regarded clinical and research activities. The third and conclusive part was composed of questions inquiring about the overall level of satisfaction with the training program.

All directors of all Italian Schools of Oncology were asked to supply a list of the trainees together with their e-mail addresses. Trainees were asked to give their consent for e-mail address disclosure. The questionnaire was published online on the website of the AIOM (http://www.aiom.it) in a reserved section and was only accessible through a direct link sent by e-mail [8]. It remained online for fifty days (from March 17^th^ 2014 to May 6^th^ 2014). After the first invitation, three further reminders were sent. The online survey was created by using Google Docs ™ online surveys maker (https://docs.google.com). Absolute frequencies and percentages were collected and organized with Microsoft Excel ^TM^.

According to the exploratory intent of the survey, no formal statistical hypothesis was prespecified, and no sample size was predefined.

In order to explore the association between the answers given to each single question and the global level of satisfaction about the training program, Chi square tests were applied. Global level of satisfaction was grouped as “Insufficient” versus “Sufficient / Good / Excellent”.

## Results

From March 17^th^ to May 5^th^ 2014, 547 Italian medical oncologist residents of 28 Oncology Medical Schools were invited to participate to the survey. A total of 311 (57%) filled in the questionnaire. Two hundred and nineteen (70%) were women and 281 (90%) were younger than 33. Participants were uniformly distributed across the five years of Oncology training. Table 1 summarizes data about residents.

**Table 1:** Data about residents.

	N	%
**Sex**		
F	219	70
M	92	30
**Age, years**		
< 33	281	90
> 33	30	10
**Year of training**		
First	61	20
Second	59	19
Third	58	19
Fourth	64	21
Fifth	69	22

### Reports about the quality of teaching

Tables 2, 3 and 4 summarize the answers about teaching.

**Table 2:** Answers about teaching (first part).

	N = 311	%	Proportion of respondents with global judgment “Insufficient”	P value
**Hours of lessons in a year**				
>30	115	37	14/115 (12%)	0.004
20–30	65	21	51/145 (26%)	
10–20	49	16		
<10	82	26		
**How frequent are the lessons**				
Weekly	76	24	8/76 (11%)	0.01
Twice a week	42	14	57/235 (24%)	
Monthly	52	17		
Twice a month	13	4		
Every 3 months	22	7		
Every 4 months	25	8		
Every 6 months	25	8		
Once a year	55	18		
**Are lessons compulsory**				
Yes	228	73	38/228 (17%)	0.002
No	83	27	27/83 (33%)	

**Table 3:** Answers about teaching (second part).

**Is it difficult to attend classes**				
Never	9	3	10/88 (11%)	0.009
Rarely	79	25		
Frequently	160	52	55/223 (25%)	
Always	63	20		
**In the five years do they repeat the same lessons**				
Yes	181	58	39/181 (22%)	0.03
No	84	27	9/84 (11%)	
N.A.	46	15	-	
**Do the lessons have diversified topics**				
Yes	131	42	10/131 (8%)	<0.0001
No	136	44	40/136 (29%)	
N.A.	44	14	-	
**Do you have an knowledge improvement**				
Not much	153	49	60/153 (39%)	<0.0001
Fairly	132	42	5/158 (3%)	
Very much	26	8		
**Do you have a good relationship with teachers**				
Yes	269	86	49/269 (18%)	0.003
No	42	14	16/42 (38%)	

**Table 4:** Answers about teaching (third part).

**Do you have opinion leaders among the teachers**				
Yes	144	46	16/144 (11%)	<0.0001
No	167	54	49/167 (29%)	
**Are there interesting topics for medical oncologists**				
Not much	188	60	62/188 (33%)	<0.0001
Fairly	107	34	3/123 (2%)	
Very much	16	5		
**Do you receive palliative care lessons**				
Yes	140	45	17/140 (12%)	0.0006
No	171	55	48/171 (28%)	
**Is there a scheduled course/teacher evaluation**				
Yes	34	11	5/34 (15%)	0.35
No	277	89	60/277 (22%)	
**Do you participate at congresses/symposia/conferences**				
Yes	273	88	49/273 (18%)	0.0006
No	38	12	16/38 (42%)	

The organization of didactic activities is not homogenous: only 228 (73%) have a compulsory attendance and 115 (37%) participants receive more than 30 hours of frontal lessons every year, while 82 (26%) receive less than 10 hours. Three hundred and two (97%) participants stated that they have some difficulties in attending lessons, because of clinical commitments.

Even the quality of classes was heterogeneous: 181 (58%) attend lessons with topics that had already been presented in previous years, 171 (55%) don’t receive classes on palliative care, 153 (49%) admit that the lessons don’t improve their knowledge. On the other hand, some classes are taught by opinion leaders and students have a high rate of participation at congresses (88%), some of which are free. Out of 273 trainees who attended conferences, 218 (80%) had attended relevant conferences (AIOM, ESMO, ASCO meetings). Two hundred and seventy-two (87%) said that it is possible to go on a training period abroad, 212 (78%) had to organize it by themselves without a predetermined path.

As reported in Tables 2,3 and 4, the proportion of students who judged insufficient their global satisfaction about training was significantly higher among those receiving less than 30 hours of lesson and less often than weekly, those without compulsory frequency to lessons and declaring difficulty to attend lessons due to clinical duties; those repeating the same lessons in the 5 years and not receiving diversified lessons according to years. Moreover, dissatisfaction was higher among students not experiencing an improvement in knowledge, having a poor relationship with teachers, not receiving lessons by opinion leaders and not receiving lessons about all the relevant topics; those not receiving lessons in palliative care and not attending to meetings and congresses.

### Perception on clinical activities

Table 5 reports the attitude towards clinical activity.

**Table 5:** Answers about clinical activity.

	N = 311	%	Proportion of respondents with global judgment “Insufficient”	P value
**Type of tumor treated**				
Only one type of tumor	65	21	14/65 (22%)	0.89
More types of tumor	246	79	51/246 (21%)	
**Is tutor present**				
Yes	240	77	40/240 (17%)	0.0007
No	71	23	25/71 (35%)	
**Do you visit with your tutor**				
Always	9	3	14/115 (12%)	0.004
Predominantly	106	34		
Called if needed	163	52	51/196 (26%)	
Never	33	11		
**Do you think you have grown in terms of professional autonomy**				
A little	50	16	20/50 (40%)	0.0003
Fairly	175	56	45/261 (17%)	
Very much	86	28		
**Do you participate to multidisciplinary groups**				
Yes	255	82	44/255 (17%)	0.0007
No	56	18	21/56 (38%)	

Surprisingly, a total of 65 (20%) trainees work in a department where only one disease is treated (e.g. breast cancer, head neck cancer, lymphoma), for all five years, even if the final qualification is not specific for a single disease. Although the Italian law requires the presence of a tutor in the hospital during all clinical activities, to enable the trainees to progressively gain independence and achieve professional growth as oncologists, this tutor is absent for 71 (23%) participants. In particular, 33 (11%) can’t contact him/her during a normal workday. In Italy, oncology medical schools residents require a training period in another clinical department in order to improve their general clinical knowledge. In detail, 96% attended an internal medicine department, while 33% attended the emergency unit, 19% cardiology. 39% spent a period in other Departments totally unrelated to Medical Oncology (e.g. allergology, rheumatology). The duration of this training varied a lot, from two months to one year.

The absence of a tutor, the lack of participation in multidisciplinary groups and the perception of a low level of professional grow correlate with a global dissatisfaction among trainees.

### Role in research activities

Trainees actively participate in research activities. One hundred and fourteen (37%) collaborate in designing clinical trials, 247 (79%) look after patients enrolled in clinical studies, 166 (53%) have an active role in study protocol management. Two hundred and nineteen (70%) actively contribute to abstract or manuscript redaction, and for 183 (84%) of them their name is listed among the authors. Table 6 summarizes the answers about research activities.

**Table 6:** Answers about research activity.

	N = 311	%	Proportion of respondents with global judgment “Insufficient”	P value
**Do you participate in the design of clinical studies**				
Yes	114	37	11 /114 (10%)	0.0002
No	197	63	54/197 (27%)	
**Do you follow patients enrolled in clinical studies**				
Yes	247	79	42/247 (17%)	0.0009
No	64	21	23/64 (36%)	
**Do you actively contribute in study conduction**				
Yes	166	53	22/166 (13%)	0.0004
No	145	47	43/145 (30%)	
**Do you actively contribute in abstracts or manuscripts redaction**				
Yes	219	70	32/219 (15%)	<0.0001
No	92	30	33/92 (36%)	
**Do you perform oral presentations**				
Yes	200	64	35/200 (18%)	0.047
No	111	36	30/111 (27%)	

Trainees who don’t participate in clinical trials design and conduction, or in abstracts and manuscripts redaction or don’t perform oral presentations are unsatisfied.

### Conclusive queries

Table 7 describes a typical daily activity. Forty-one (14%) students spend less than 1 hour per day engaged in clinical activities, 137 (44%) spend more than two hours per day writing patients’ clinical reports, 48 (15%) spend more than 2 hours per day entering clinical data in AIFA (Agenzia Italiana del Farmaco, Italian Drug Agency) registry, a national system that controls and manages drug reimbursements. Forty-six (15%) are busy in filling in case report forms (CRF) for more than 2 hours per day.

**Table 7:** Details on daily activities.

	0	Less than 1 hour	2–4 hours	More than 4 hours
**Clinical activity**	2 (1%)	41 (13%)	152 (49%)	116 (37%)
**Chemotherapy management**	20 (6%)	85 (27%)	145 (47%)	61 (20%)
**Write medical records**	1 (1%)	70 (23%)	169 (54%)	71 (23%)
**Write patient's clinical report**	9 (3%)	165 (53%)	109 (35%)	28 (9%)
**Enter clinical data in AIFA register**	63 (20%)	200 (64%)	41 (13%)	7 (2%)
**Research activity**	95 (31%)	124 (40%)	76 (24%)	16 (5%)
**Fill in CRF**	133 (43%)	132 (42%)	37 (12%)	9 (3%)
**Personal study**	67 (22%)	187 (60%)	49 (16%)	8 (3%)

Participants were required to express a global judgment on satisfaction about teaching, clinical and research activities. One hundred and ninety-five (63%) expressed a low level of satisfaction for teaching and 167 (54%) for research activity; 269 (86%) stated a good opinion about the organization of clinical activities. Regarding the question about the global judgment of Medical Oncology School, 246 (79%) of trainees gave a positive global judgment.

## Discussion

Medical Oncology is recognized as an independent specialty in many countries [2], but training programs differ from one University to another, even in the same country; in the literature no studies focused on the training of medical oncology residents. The quality of training programs is essential for the formation of properly qualified oncologists, and even if ASCO/ESMO provided a set of guidelines for a global curriculum in Medical Oncology, these are still far from being applied in a systematic way [3]. One of the aspects that could be analyzed in order to directly assess the quality of a specialty school is to explore the residents’ point of view. Using an online survey, the present study provides a comprehensive overview of teaching, clinical and research activities carried out by medical oncology students in Italy. This study also considers and assesses the global levels of student satisfaction with the delivered training.

In the literature, few papers explored the opinion of residents and directors of courses of the Hematologic/Oncologic area on the quality of training, formation program and training organization [9–12]. In 2006, Semrau et al. conducted a survey among German Radiation Oncologist residents, with the aim to evaluate residents satisfaction with their training [11]. At the same time, the DEGRO (German Society of Radiation Oncology) published training guidelines for radiation oncologists, including recommendations for content and organization of learning courses [13]. In our experience, the idea to perform a survey was developed by a group of oncologists and residents, all AIOM members. We felt the need to involve the scientific society to better understand the real situation of the organization of the schools, which are now almost exclusively managed by the universities. The high response rate achieved (57%), even among students attending the later years of training, denotes the remarkable interest in the topic of the survey.

The majority of participants (70%) were women. In recent years, an increasing presence of women has been observed in the overall medical profession and, in particular, specialties such as pediatrics, obstetrics-gynecology, and internal medicine [14]. In Italy, the test for admission to the Italian school of medicine is mainly passed by female students [15,16], probably because of their attitude to face a meticulous study. As expected, 90% of participants were younger than 32 years of age. Indeed, the average age of people getting a medical degree in Italy is 26.7 [15], and the subsequent oncology medical school follows the European model of a 5-year curriculum [3]. As indicated by the Italian Ministry of Education, University and Research (MIUR), teaching and clinical activities of oncology medical schools should follow the rules and the programs suggested by the councils of each school before the beginning of the academic year [17]. This explains the differences found in didactic activities in terms of frequency, duration and compulsoriness.

However, it is certainly alarming and intolerable that the majority of participants (72%) encounter serious difficulties in attending classes during the academic years. In fact, residents argued that they are often involved in full time clinical activities and this limits their participation in lessons. Moreover, almost a half of the students (49%) didn’t find the lessons useful for the learning process and with a considerable lack of interesting topics (60%). This severe opinion might be due to the high rate of repeated lessons through the five years, with 58% of students admitting topic similarities in the 5 teaching years.

As recommended by ESMO and ASCO, Oncology residents should be skilled in the comprehensive management of patients with different neoplastic diseases on an in-patient and out-patient basis, for both acute and chronically ill patients in order to learn the natural history of cancer and the effectiveness of the various therapeutic programs [3]. Our findings suggest that oncologist trainees in Italy spend most of the school time in clinical or clinical-related activities, even in multidisciplinary teams, that allows them to acquire high medical skills. Nevertheless, 1/5 of the students are trained only in one type of disease or cancer area even if the final qualification is not specific for a single disease. This organization results in Oncologists who are not skilled to work in all the different conditions of the hospitals in our Country. In fact, only few Italian patients are cured in referrals Centers, where oncology departments are organized according to pathology units (e.g. breast unit, lung unit). The majority of them are cured in peripheral hospitals, where every single Oncologist has to manage many different malignancies.

Half of the students interviewed answered that they have never received lessons in supportive and palliative care. Popescu et al. recognized that a well-trained medical oncologist should be prepared even in supportive, palliative and end of life care [2]. In the literature, there are some examples regarding the lack of attention to palliative care: Thomas et al., through a survey conducted among the US Hematology/Oncology residents, showed that many of them are inadequately prepared to manage patients at the end of life [18]. Mougalian et al. stated that higher-quality teaching, particularly in palliative care, is associated with less burnout among residents. In fact, lower emotional exhaustion scores were associated with the residents' perception of having received better teaching about certain end-of-life topics [19].

In our study we showed that some students spend a significant part of their working day completing CRF and AIFA report forms (Table 7), tasks that are relevant for the overall management of the unit, but that should not be a core task for the student in training.

Finally, medical oncology students are strongly encouraged to gain experience in clinical and/or translational cancer research as part of their training. Our survey confirmed that the majority of oncologist trainees are engaged in research activities, which also include the redaction of scientific manuscripts or oral presentations at seminars or conferences. 88% stated that they are allowed to participate to meetings, most of them with national/international relevance. This good point is probably due to a well-established collaboration between oncology institutions and pharmaceutical companies who fund cultural efforts.

Some studies explore the well-being and burnout levels of residents [7] and of young oncologists [6]. Interestingly, Banerjee explains that her survey has shown that burnout is a common problem for young oncologists, and this is likely due to the mental and emotional load of work activities, because oncologists have to “…*make complex decisions about cancer management*, *supervise the use of toxic therapies*, *work long hours*, *and continually face patients suffering and dying*”.

Even if burnout levels were not assessed in our survey, in the final part of the questionnaire some conclusive queries about the level of satisfaction were made. Suboptimal levels of satisfaction were recorded mostly regarding didactical activities and this is surely due to the difficulties for many students interviewed to take part in classes, because of clinical commitments, but also to an overall poor perceived utility of lessons. It's very surprising to learn that half of the interviewed students stated that lessons didn't improve their knowledge.

In conclusion, this survey summarized the opinions about the School of Medical Oncology of the Italian Oncology residents. There is an overall attention to this topic, demonstrated by the high percentage of participants among students of all five years of the course. The global satisfaction was higher for clinical activities than for research and didactical activities. Greater effort should be made to improve the didactical content and organization. The introduction of an evaluation questionnaire in every single school could probably help teachers in identifying the weaknesses in order to improve the quality of training, which could be the first step in empowering qualified Oncologists.
